# Correlates of low resilience and physical and mental well-being among Black youths in Canada

**DOI:** 10.1186/s12889-024-19440-7

**Published:** 2024-08-31

**Authors:** Folajinmi Oluwasina, Jo Henderson, Kwame McKenzie, Delores V. Mullings, Andre M.N Renzaho, Tolulope Sajobi, Cecile Rousseau, Ambikaipakan Senthilselvan, Hayley Hamilton, Bukola Salami

**Affiliations:** 1 University of Alberta, Edmonton, Canada; 2 University of Toronto, Toronto, Canada; 3 Memorial University, Newfoundland, Canada; 4 Western Sydney University, Penrith, Australia; 5 University of Calgary, Calgary, Canada; 6 McGill University, Montreal, Canada; 7 Centre for Addiction and Mental Health, Toronto, Canada; 8 Red Deer Polytechnic, Red Deer, Canada

**Keywords:** Young adult, Employment, Resilience, Mental health, Canada

## Abstract

**Background:**

Resilience has gained considerable attention in the mental health field as a protective factor that enables individuals to overcome mental health issues and achieve positive outcomes. A better understanding of resilience among Black youth is important for supporting the strengths and capacities within this population. This study seeks to investigate the correlates of resilience among Black youths in Canada.

**Methods:**

The survey was conducted online through REDCap between November 2022 and March 2023. The Brief Resilience Scale (BRS) was utilized to measure the capacity of participants to recover from or bounce back from stress. The BRS comprises six five-point Likert scale items. Data were analyzed employing a bivariate analysis followed by a multivariable binary logistic regression.

**Results:**

A total of 933 Black youths participated in the study across all Canadian provinces, of which 51.8% (483) identified as female and 46.7% (436) as male. Most respondents 51.3% (479) were between the ages of 16 and 20 years, with 28% (261) between the ages of 21 and 25 years, and 20.2% (188) between the ages of 26 and 30 years. In terms of employment, 62.0% (578) were working part-time, 23.7%, (220) were unemployed, and 9.8% (91) were working full-time. Over a third of participants (39.3%, 331) rated their mental health over the last month as good, with 34% (317) giving a rating of poor and 20.9% (195) giving a rating of fair. Black youths who were working part-time had four times greater odds of expressing low resilience (OR: 4.02; 95% CI: 1.82–11.29) than those who were not working. Black youth who ranked their mental health as poor were about nine times (OR: 8.65; 95% CI: 1.826–21.978) more likely to express low resilience.

**Conclusion:**

In this study, the Black youth participants reported relatively low resilience scores. Employment, physical health, and mental health status were factors that contributed to low resilience. Further studies are needed to examine the causal link between resilience and its dynamic effect on health outcomes among Black youth. More interventions are needed to make mental health services accessible to Black youth in a more culturally sensitive way with cross-culturally trained professionals.

## Introduction

Resilience refers to an individual’s ability to adapt to adversity, trauma, or significant life stressors and recover. Resilience also plays a significant role in encouraging positive mental well-being and reducing the risk of mental health disorders among different populations [1]. Resilience is predicated on the assumption that individuals have previously confronted unfavourable life situations and can leverage these experiences to effectively modify their behaviour [2–4]. It is imperative to acknowledge that an individual may have resilience in a specific domain while lacking it in another; for instance, youth may exhibit satisfactory academic performance while concurrently experiencing symptoms of worry or depression [5, 6]. This implies the process of adaptation is influenced, at least in part, by the surrounding context.

Several studies [7–9] have used different terminologies to characterize the three resilience models, which essentially explain the same stress-related mechanisms for quality adaptation. They include the compensating model, the challenge model, and the immunity vs. vulnerability model’s protective factor. According to O’Leary [7], the protective factor model of resilience describes how protection and risk factors interact to lower the likelihood of a bad result and attenuate the impact of risk exposure. This resilience model is based on systems theory and developmental literature. It suggests that despite adverse or unpleasant life conditions, these protective variables promote positive results and healthy personality traits [8, 9]. The ability to recover self-esteem, academic and professional skills, intrapersonal reflecting skills, emotional management abilities, planning abilities, life skills, and problem-solving abilities were among the protective factors found [9].

Generally, individuals in their youth have a range of psychological, emotional, and behavioural difficulties, particularly as they navigate the transitional periods between childhood, adolescence, and early adulthood [10]. Several factors, such as multidimensional hurdles or life events, may expose young people to the possibility of acquiring a sense of discontentment with life. This, in turn, can result in significant health consequences. In the general population of youth, normal to higher levels of resilience are consistently associated with improved physical and mental health [11, 12], with resilience possibly serving as a protective factor against the onset of chronic illness or depression [13]. These findings suggest a potential causal association between higher levels of resilience and general well-being. A study focusing on young and older adults found evidence that resilience can be developed or maintained notwithstanding poor physical well-being [14]. Such findings suggest that resilience may be utilized to enhance the health of youths and older populations.

Black youth are a unique population that often faces persistent stressors and other obstacles to resilience in the context of distinctive and complex issues. Black youth confront social, economic, and systemic challenges that might harm their mental health and well-being [15]; for instance, they face more discrimination, racism, social inequality, and restricted resources and opportunities than their white counterparts. According to Wyatt et al. [16], Black youth are disproportionately affected by mental health illnesses, including post-traumatic stress disorder, anxiety, and depression. These challenges might impact Black youth’s academic achievement, social life, and overall quality of life. Black youth tend to adopt fearlessness, loss, or suppression of fear emotions in response to chronic and unpredictable trauma exposure and unresponsive support systems. However, life contentment can eradicate the symptoms of depression and generalized anxiety disorder (GAD) and boosts self-respect, confidence, and physical and psychological health among young people [17, 18]. Resilience also increases self-discipline [19, 20] and promotes youth well-being and life satisfaction [21, 22]. Indeed, people with higher levels of resilience have fewer psychological issues [23]; a similar relationship has been found between resilience and physical health. Despite the fact Black youths are disproportionately exposed to stress that can increase their vulnerability to physical and mental health difficulties, existing mental health interventions fail to sufficiently cater to their specific needs [24]. Black youth mental health treatment drop-out rates are high, and many standard treatments are less effective for Black youth than for other groups [24]. Salami et al. [25] noted their Black youths participants held the belief that they are immune to mental health disorders and anticipated overcoming adversities through resilience.

Black youth physical and mental health is a major public health issue because it affects their development and life paths [26]. This group faces unique adversities, such as systemic racism, social inequalities, discrimination, and limited access to resources, which can negatively impact resilience, physical health, and mental health [27, 28]. Black youth with stronger resilience may have better emotional regulation, problem-solving, and adaptive resilience skills, which in turn results in better mental health [29]. Physical health is also an important part of well-being, and inequities in health outcomes among Black youth have drawn attention in recent years [30].

A better understanding of the impact of resilience on the physical and mental health of Black youth can inform targeted interventions and health promotion [31]. The impact of systemic barriers is also important as these may be significant factors affecting the resilience, physical health, and mental health of Black youths [32]. As such, this study aimed to assess the correlates and predictors of resilience and well-being of Black youths in Canada.

## Methods

### Study setting and sample size estimation

This study was conducted with Black youth living in Canada. According to the Canadian census (2021), a total of 1,547,870 Canadians identify as Black, constituting 4.3% of the total population [33]. Data were collected using a proportionate-to-size sampling approach, which ensures the selection of a representative sample of Black youth from all 10 provinces and three territories of Canada. Black Youths who are between the ages 16 to 30 years, and who have lived in Canada for more than two years were included in the study. For our analysis, a 95% confidence interval and a margin of error of ± 3% required a sample size of 933.

### Study design and institutional review board approval

This descriptive cross-sectional study employed self-administered, anonymous, online questionnaires. Respondents who self-identified as Black youth living in Canada participated. The study was conducted in accordance with the provisions of the Declaration of Helsinki (Hong Kong Amendment). All participants were provided with an online information leaflet and informed consent was obtained before participation. The study received approval from the Health Ethics Research Board of the University of Alberta (Ethics ID: Pro00116630).

### Data collection and outcome measures

The online survey was used to collect data from young Black people between the ages of 16 and 30 years who were living in Canada. This study utilized Research Electronic Data Capture (REDCap) electronic data capture tools for data collection and management. REDCap offers a user-friendly interface for capturing verified data, maintains an audit trail to monitor data manipulation and export activities, provides automated measures for continuous data downloads to popular statistical packages, enables data integration as well as compatibility with external sources, and ensures the security of data through its secure infrastructure [34, 35]. The data collection instruments used in this study drew upon relevant literature and previously validated instruments. The primary variables of interest were pertinent demographic data including age, gender, sexual orientation, education, and income as well as the Likert scale rating their physical and mental well-being including rating and resilience.

The standardized measure used to evaluate resilience was the Brief Resilience Scale (BRS), which assesses an individual’s ability to bounce back or recover from stress [36]. The BRS consists of six items measured on a five-point Likert scale. Responses are summed to provide a total score ranging from 6 to 30, from which the mean score is then calculated. A mean score from 1.00 to 2.99 indicates low resilience, from 3.00 to 4.30 shows normal resilience, and from 4.31 to 5.00 indicates high resilience [36]. For analysis purposes considering a binary logistic regression, the scores were recategorized into two groups: normal to high resilience (≥ 3) and low resilience (< 3). The literature shows the BRS has good internal consistency, with Cronbach alphas ranging from 0.80 to 0.90 and test-retest reliability coefficients for a two-week interval of 0.61 to 0.69. A single-item measure of self-rated mental health (SRMH) was used in the assessment of physical and mental well-being. The item asks respondents to rank their mental health on a five-point scale (good to poor). In a study conducted by Fung et al., [37] it was found that the single-item SRMH measure is a reliable tool that is positively linked to self-esteem. However, it is negatively associated with common mental health symptoms such as depression and PTSD symptoms, as well as self-reported psychiatric treatment consumption. A subgroup of participants completed the retest after an average of 9.32 days (SD = 3.97). The single-item measure of SRMH showed moderate to good test-retest reliability with an ICC score of 0.75 (95% CI: 0.65–0.82, *p* < 0.001). These findings suggest that the single-item SRMH measure can be used as a public health measure to assess self-perceived general mental health. It reflects one’s overall mental well-being and correlates with other mental health conditions such as depression and anxiety, as demonstrated in this study.

### Statistical analysis

Data analysis was performed using SPSS [38]. Demographic characteristics of Black youth, as well as responses to questions related to physical and mental well-being, were summarized by absolute numbers and percentages. Only completed responses were reported, with no data imputation. Chi-square and Fisher’s exact tests with two-tailed significance (*p* ≤ 0.05) were conducted to examine the relationship between the demographic characteristics of Black youth and responses to questions related to physical and mental well-being. The demographic features of Black youths, along with their responses regarding physical and mental well-being, were presented in a concise manner using both absolute figures and percentages. The reported responses primarily consisted of completed data without any instances of data imputation. Analysis was performed using the chi-square or Fisher’s exact test, with a two-tailed significance level set at *p* ≤ 0.05. The focus of this analysis was to examine the relationship between the socioeconomic background of Black youth and their responses to questions regarding their physical and mental well-being.

The variables that showed significance at the *p* ≤ 0.1 and approaching significance level in the bivariate analysis were included in the multivariable binary logistic regression analysis. This analysis aimed to investigate the probability of respondents exhibiting low resilience. The association between the predictor variables and the likelihood of low resilience was assessed using odds ratios. The analysis accounted for potential confounders in multiple logistic regression.

## Results

A total of 933 Black youth participated in this survey. Table 1 shows the distribution of the socio-demographic characteristics of the participants. Just over half of the respondents (51.8%, 484) were between the ages of 16 and 20 years, with 28% (261) aged 21 to 25, 20.2% (188) aged 26 to 30, and 0.5% (5) below the age of 16. Slightly more respondents identified as female (51.8%, 483) than male (46.7%, 436). The vast majority (95.8%, 894) identified as straight/heterosexual. In terms of relationship status, 61.7% (576) self-reported as single, with 21.3% (143) in either a committed relationship or dating and 15.3% (143) as married/common law. The predominant religious affiliations were Christian (87.5%, 816) and Muslim (9.3%, 87). Most respondents were either full-time (68.5%, 639) or part-time (17.0%, 159) students. Most were either pursuing (39.0%, 364) or had completed (14.6%, 136) a university degree, while others had a college certificate or diploma in progress (18.4%, 172) or completed (8.8%, 82). Most respondents (62.0%, 578) were working part-time, with others unemployed (23.7%, 220) or employed full-time (9.8%, 91). Most (63.7%, 594) reported earning less than $40,000 per year, with 18.3% (171) earning between $40,001 and $60,000 per year followed by smaller percentages for higher income brackets.

**Table 1:** Socio-demographic characteristics of the respondents (*n* = 933)

Characteristics	Categories	Frequency (%)
Age	16–20	484 (51.8)
	21–25	261 (28.0)
	26–30	188 (20.2)
Gender	Male	436 (46.7)
	Female	483 (51.8)
	Non-binary	4 (0.4)
	Trans-gendered	1 (0.1)
	Gender-fluid	2 (0.2)
	Genderqueer	5 (0.5)
	Transwoman	1 (0.1)
	Prefer not to answer	1 (0.1)
Sexual orientation	Straight/heterosexual	894 (95.8)
	Lesbian	14 (1.5)
	Gay	8 (0.9)
	Bi-sexual	10 (1.1)
	Queer	5 (0.5)
	Prefer not to answer	1 (0.1)
Relationship	Married	143 (15.3)
	Committed relationship	199 (21.3)
	Single	576 (61.7)
	Divorced	9 (1.0)
	Widowed	1 (0.1)
	Other	5 (0.5)
Religion	Christian	816 (87.5)
	Muslim	87 (9.3)
	Not religious	30 (3.2)
Student status	No	135 (14.5)
	Yes- part-time	159 (17.0)
	Yes- full-time	639 (68.5)
Employment	Full-time	91 (9.8)
	Part-time	578 (62.0)
	Unemployed	220 (23.7)
	Volunteering	3 (0.3)
	Student	37 (4.0)
Education status	High school or less	39 (4.2)
	College certificate or diploma in progress	172 (18.4)
	College certificate or diploma completed	82 (8.8)
	University degree in progress	364 (39.0)
	University degree completed	136 (14.6)
	Postgraduate degree in progress	102 (10.9)
	Postgraduate degree completed	37 (4.0)
	Other	1 (0.1)
Annual income	< $40,000	594 (63.7)
	$40,001 – $60,000	171 (18.3)
	$60,001 – $80,000	79 (8.5)
	$80,001 – $100,000	31 (3.3)
	$100,001 – $150,000	36 (3.9)
	> $150,001	22 (2.4)

Table 2 shows the summary statistics of physical and mental well-being for the Black youth participants. Approximately 44% (377) of respondents rated their physical health over the last month as good, with 36.4% (307) giving a rating of poor and 18.9% (159) as fair. Less than half of the participants (39.3%, 331) rated their mental health over the last month as good, with 34% (317) giving a rating of poor and 20.9% (195) as fair. The largest proportion of respondents (42.2%, 406) reported their average duration of sleep per night was 7–8 h, with smaller proportions reporting less sleep (4–6 h: 17.9%, 151; < 4 h: 12%, 105) or more sleep (> 10 h: 14%, 119; 9–10 h: 7.4%, 62).

**Table 2:** Self-reported physical and mental well-being variables for the respondents (*n* = 933)

Variable	Good *N* (%)	Fair *N* (%)		Poor *N* (%)	
Rate your physical health over the last month	377 (44.7)	159 (18.9)		307 (36.4)	
Rate your mental health over the last month	331 (39.3)	195 (20.9)		317 (34.0)	
Variable	< 4 h	4–6 h	7–8 h	9–10 h	> 10 h
Currently, how many hours per night do you sleep on average?	105 (12.5)	151 (17.9)	406 (42.2)	62 (7.4)	119(14.1)

Table 3 illustrates the results of the bivariable analysis (p-values from chi-square and Fisher’s exact test) of the association between the predictor variables and resilience. The prevalence of low resilience among study participants was 60.7% (558/919). Eight variables that were statistically significant at *p* < 0.05 (*Relationship status; Religion; Annual income; How long have you lived in Canada?; Which language do you speak at home?; Rate your physical health in the last one month; Rate your mental health in the previous month; How many hours per night do you sleep on average per day?*) and one at *p* < 0.1 (*Are you currently a student*?) were selected for the multivariable binary logistic regression model (Table 4).

**Table 3:** Bivariate analysis showing the relationship between variables and respondents’ likelihood of low resilience

Characteristics		Low resilience	High resilience	Chi-square value	P- value
		n (%)	n (%)		
Age	16–20	282 (60.1)	187 (39.9)	4.22	0.24
	21–25	167 (64.7)	91 (35.3)		
	26–30	105 (56.1)	82 (43.9)		
Gender	Male	243 (57.4)	180 (42.6)	8.44	0.29
	Female	303 (62.9)	179 (37.1)		
	Non-binary	3 (75.0)	1 (25.0)		
	Transgendered	1 (100.0)	0 (0.0)		
	Gender fluid	1 (50.0)	1 (50.0)		
	Genderqueer	5 (100.0)	0 (0.0)		
	Transwoman	1 (100.0)	0 (0.0)		
	Prefer not to answer	1 (100.0)	0 (0.0)		
Sexual orientation	Straight/heterosexual	532 (60.5)	348 (39.5)	5.62	0.34
	Lesbian	9 (64.3)	5 (35.7)		
	Gay	4 (50.0)	4 (50.0)		
	Bi-sexual	7 (70.0)	3 (30.0)		
	Queer	5 (100)	0 (0.0)		
	Prefer not to answer	0 (0.0)	1 (100.0)		
Relationship status	Married	89 (62.7)	53 (37.3)	15.09	**0.01**
	Committed	99 (50.5)	97 (49.5)		
	Single	361 (63.7)	206 (36.3)		
	Divorced	5 (55.6)	4 (44.4)		
	Widowed	0 (0.0)	1 (100)		
	Others	4 (100)	0 (0.0)		
Religion	Christian	484 (59.2)	333 (40.8)	6.58	**0.03**
	Muslim	60 (70.9)	22 (26.8)		
	Not religious	14 (70.0)	6 (30.0)		
Are you currently a student?	No	82 (60.7)	53 (39.3)	5.45	0.07
	Yes-Part-time	103 (69.1)	46 (30.9)		
	Yes-Full-time	373 (58.7)	262 (41.3)		
Employment	Full-time	47 (51.6)	44 (48.4)	4.11	0.39
	Part-time	343 (60.8)	222 (39.2)		
	Unemployed	137 (62.3)	83 (37.7)		
	Volunteering	2 (66.7)	1 (33.3)		
	Student	25 (67.6)	12 (32.4)		
Educational status	High sch.	22 (56.4)	17 (43.6)	4.16	0.76
	College cert/dipl. in progress	99 (60.7)	64 (39.3)		
	College cert/dipl completed	45 (55.6)	36 (44.4)		
	University degree in progress	227 (62.9)	134 (37.1)		
	University degree completed	78 (57.8)	57 (42.2)		
	Postgrad. degree in progress	64 (62.7)	38 (37.3)		
	Postgrad. degree completed	23 (62.2)	14 (37.8)		
	Other	0 (0.0)	1 (100.0)		
Annual income	<$40,000	379 (65.0)	204 (35.0)	22.40	**< 0.00**
	$40,001 – $60,000	100 (58.2)	69 (40.8)		
	$60,001 – $80,000	34 (43.0)	45 (57.0)		
	$80,001 – $100,000	11 (36.7)	19 (63.3)		
	$100,001 – $150,000	21 (58.3)	15 (41.7)		
	>$150,001	13 (59.1)	9 (40.9)		
How long have you lived in Canada?	Less than a year	22 (61.1)	12 (38.9)	18.43	**0.01**
	One to two years	54 (59.9)	48 (47.1)		
	Three to four years	99 (63.5)	57 (36.5)		
	Six to ten years	46 (61.3)	29 (38.7)		
	Eleven to fifteen years	75 (77.3)	22 (22.7)		
	Sixteen years or more	51 (65.4)	27 (34.6)		
	Born in Canada	209 (56.0)	164 (44.0)		
What language do you speak at home?	English	350 (59.8)	235 (40.2)	5.82	**0.03**
	French	68 (71.6)	27 (28.4)		
	Somali	24 (66.7)	12 (33.3)		
	Yoruba	13 (72.2)	5 (27.8)		
	Swahili	28 (41.2)	40 (58.8)		
	Twi	37 (71.2)	15 (28.8)		
	Others	38 (58.5)	27 (41.5)		
How would you rate your physical health over the last month?	Good	315 (49.2)	325 (50.8)	5.34	**< 0.00**
	Fair	106 (80.9)	25 (19.1)		
	Poor	60 (84.5)	11 (15.5)		
How would you rate your mental health over the last month?	Good	70 (21.1)	261 (78.9)	3.75	**< 0.00**
	Fair	106 (54.4)	89 (45.6)		
	Poor	305 (96.5)	11 (3.5)		
How many hours per night do you sleep on average per day?	< 4 h	69 (65.7)	36 (34.3)	3.51	**< 0.00**
	4–6 h	150 (100.0)	0 (0.0)		
	7–8 h	97 (23.9)	309 (76.1)		
	9–10 h	46 (74.2)	16 (25.8)		
	> 10 h	119 (100.0)	0 (0.0)		

*p* < 0.05

The regression model comprised of 9/14 chi-square variables that predicted with one variable demonstrating marginal significance after removing six variables.

The logistic regression model *Χ*^2^ of 30.95 (df = 8; *n* = 919) was statistically significant (*p* < 0.001), indicating the regression model could differentiate between Black youths who likely have low or normal to high resilience. The statistical model explained a range of variance from 15.1% (Cox and Snell *R*^2^) to 24.8% (Nagelkerke *R*^2^). A Hosmer-Lemeshow goodness-of-fit test indicated the model was properly fitted (χ^2^ = 5.59; *p* = 0.99). Additionally, the model accurately classified 97.1% of the cases. No variables had a strong connection to other variables (r_s_ > 0.7). The adjusted odd ratio controls for other predictor variables in the model.

Overall, Black youths who were working part-time had four times greater odds of expressing low resilience (OR = 4.02; 95% CI: 1.82–11.29) than those who were not working. Black youths who rated their physical health as fair over the last month had seven times greater odds (OR = 7.05; 95% CI: 3.715–14.29) of expressing low resilience than Black youths who rated their physical health as good. Black youths who rated their physical health as poor over the last month had 12 times greater odds of expressing low resilience (OR = 12.41; 95% CI: 1.106–15.385) than respondents who rated their physical health as good. Black youths who ranked their mental health as poor in the last month had more than eight times greater odds (OR = 8.65; 95% CI: 1.826–21.978) of expressing low resilience than those who rated their mental health as good.

**Table 4:** Multivariable logistic regression model predicting the presence of low resilience among black youth

Variables in equation	*P* value	Odds ratio	95% CI for odds ratio	
			Lower	Upper
**What best describes your current relationship status?**				
Married/common-law	0.547			
Committed/dating	0.257	0.309	0.040	2.358
Single	0.638	0.629	0.091	4.346
Divorced	0.265	5.506	0.275	110.298
Widowed	1.000	0.008	0.000	3.234
Others	0.999	0.000	0.000	6.462
**Are you currently a student?**				
No	0.042			
Yes- Part-time	0.012	4.021	1.820	11.297
Yes- Full-time	0.467	0.611	0.162	2.308
**What is your religion?**				
Christian	0.467			
Muslim	0.484	0.539	0.096	3.035
Not religious	0.302	0.160	0.005	5.183
**How long have you lived in Canada?**				
< 1 year	0.375			
1–2 years	0.637	0.447	0.016	12.577
3–5 years	0.298	0.175	0.007	4.654
6–10 years	0.622	0.463	0.022	9.828
11–15 years	0.317	0.228	0.013	4.135
16 years or more	0.024	0.008	0.000	0.526
Born in Canada	0.182	0.162	0.011	2.341
**What is your annual income?**				
<$40,000	0.765			
$40,001 – $60,000	0.446	2.099	0.312	14.144
$60,001 – $80,000	0.520	0.475	0.049	4.586
$80,001 – $100,000	0.295	5.968	0.211	168.857
$100,001 – $150,000	1.000	5.088	0.000	9.673
>$150,001	0.995	4.519	0.000	3.465
**What language(s) do you usually speak at home?**				
English	0.393			
French	0.043	5.187	1.142	9.365
Somali	0.317	0.358	0.048	2.679
Yoruba	0.993	0.000	0.000	4.564
Swahili	0.400	2.670	0.271	26.296
Twi	0.908	0.790	014	43.100
Others	0.490	2.022	0.274	14.926
**How would you rate your physical health over the last month?**				
Good	0.017			
Fair	0.032	7.055	3.715	14.293
Poor	0.013	12.410	1.106	15.385
**How would you rate your mental health over the last month?**				
Good	0.010			
Fair	0.433	0.629	0.198	2.002
Poor	0.007	8.651	1.826	21.978
**Currently**,** how many hours per night do you sleep on average?**				
< 4 h	1.000			
4–6 h	0.987	0.000	0.000	1.462
7–8 h	0.994	0.000	0.000	3.893
9–10 h	0.989	0.000	0.000	5.378
> 10 h	0.986	0.000	0.000	1.264
**Constant**	**0.989**	**28.000**		

## Discussion

The findings of this study provide evidence of significant correlations between resilience, physical health, and mental health among Canadian Black youths. This study identified the type of employment as a predictor of low resilience among Black youth, with those working part-time being four times more likely to experience low resilience than Black youths who were not working. This finding is in line with studies with youth that report a significant interaction between type of employment, job demand, and resilience [39, 40]. Individuals exhibiting lower levels of resilience experience more unfavorable psychological and work-related outcomes, particularly in work environments characterized by low levels of pressure [41]. Part-time work can increase stress and strain, affecting resilience and coping. A study on race, risk, and resilience and Black youth reported higher levels of racial socialization and well-being affected educational and employment levels, which in turn resulted in low levels of resilience; the study further indicated that 44% of its participants reported suffering from either physical and/or mental health issues [42]. The higher risk of inadequate resilience among Black youth in the present study who worked part-time confirms that employment status affects resilience outcomes for this population.

Self-rated physical health and resilience among Black youths were also strongly correlated. Black youths who rated their physical health as fair or poor over the last month were far more likely (seven times and 12 times, respectively) to experience low resilience than Black youths who rated their physical health as good in the last month. This aligns with research that demonstrates physical health affects resilience outcomes [43, 44]. Black youths with fair or poor physical health may struggle to cope, which can lower resilience. To overcome hurdles and promote holistic well-being, resilience-building initiatives should take Black youth’s physical health into consideration.

Black youth who ranked their mental health as poor in the last month were notably (nine times) more likely to experience low resilience compared to those who rated their mental health as good. This relationship between mental health status and resilience has also been noted in previous studies [29, 44, 45]. Poor mental health reduces resilience to stress and adversity. Studies with youth show they are more likely to have lower community resilience if under 25 years of age [45]; this group can also exhibit considerable psychological impairments in response to disasters, with limited capacity for resilience compared to older adults [46].

Overall, the findings from this study are consistent with other literature on resilience in youth and well-being, including those with varied populations. A systematic review on this topic noted comparable connections between resilience, employment status, physical health, and mental health for racially and ethnically diverse youth [47]. Such consistent findings across populations show the observed correlations are generalizable.

### Study limitations

Data for this study were obtained through online surveys and cannot describe the entire population. Convenience sampling was used and biased respondents may self-select into the sample. This study may have had different results if it had been conducted before the COVID-19 pandemic, which may have exposed participants to additional stress. Additionally, a unidimensional measure was used to analyze resilience in this study. The use of a multidimensional scale that distinguishes the roles of family and peers as protective factors linked with resilience may influence the overall score.

## Conclusions

This study showed employment and physical and mental well-being contribute to low resilience among Black youth in Canada. Substantial relationships were noted between resilience and health outcomes, highlighting the need to understand and foster resilience in this vulnerable group. Black youth who worked part-time had much lower resilience than those who did not work. This underscores the need for supportive workplaces and skill development to build resilience among Black youths.

The relationship between self-rated physical health and resilience in Black youth was also clear. Low resilience was linked to fair or poor physical health. To improve resilience and well-being, Black adolescents should address physical health inequities and adopt healthy lifestyles. Not surprisingly. mental health and resilience were also linked in this population. Black youths with poor mental health had much less resilience. These findings emphasize the need for mental health support structures and resources in the Black community. Overall, our findings demonstrate that resilience is influenced by employment position, physical health, and mental well-being. These findings support resilience research on youth and emphasize the urgent need for holistic health programs that address both physical and mental well-being.

This study has important implications for policymakers, healthcare professionals, and community organizations trying to promote Black youth health in Canada. Understanding resilience variables helps create strategies and programs that support this population and improve health. This study sheds light on Black youth resiliency and physical and mental health in Canada. Initiatives to promote holistic well-being and resilience-building should consider physical health as well as mental health. The findings also emphasize the need for customized therapies that address this population’s particular issues. Improving resilience and addressing health determinants can improve the well-being of Black youth towards a more equal and inclusive society. Further studies are needed to examine the causal link between resilience and its dynamic effect on health outcomes among Black youth and also a need to understand more about Black youth’s experience in the workplace with connections to economic challenges.

## Data Availability

The datasets generated and/or analyzed during the current study are not publicly available due to the nature of the data collected for this study. The data collected involves sensitive information related to the well-being of Black youths in Canada. Ensuring the privacy and confidentiality of the study participants is of utmost importance. Sharing the raw data could compromise the anonymity and confidentiality guaranteed to the participants during the research process. Therefore, withholding the data is a measure taken to uphold ethical standards. Data will be available upon reasonable request from the corresponding author.
